# Loss of α-Calcitonin Gene-Related Peptide (αCGRP) Reduces Otolith Activation Timing Dynamics and Impairs Balance

**DOI:** 10.3389/fnmol.2018.00289

**Published:** 2018-08-24

**Authors:** Sherri M. Jones, Sarath Vijayakumar, Samantha A. Dow, Joseph C. Holt, Paivi M. Jordan, Anne E. Luebke

**Affiliations:** ^1^Department of Special Education and Communication Disorders, University of Nebraska, Lincoln, NE, United States; ^2^Department of Neuroscience and Del Monte Institute for Neuroscience, University of Rochester Medical Center, Rochester, NY, United States; ^3^Department of Otolaryngology, University of Rochester Medical Center, Rochester, NY, United States; ^4^Department of Biomedical Engineering, University of Rochester Medical Center, Rochester, NY, United States

**Keywords:** CGRP, otolith, vestibular efferent, sensory coding, mouse, VsEP, rotarod, balance

## Abstract

Calcitonin gene-related peptide (CGRP) is a neuroactive peptide that is thought to play a role at efferent synapses in hair cell organs including the cochlea, lateral line, and semicircular canal. The deletion of CGRP in transgenic mice is associated with a significant reduction in suprathreshold cochlear nerve activity and vestibulo–ocular reflex (VOR) gain efficacy when compared to littermate controls. Here we asked whether the loss of CGRP also influences otolithic end organ function and contributes to balance impairments. Immunostaining for CGRP was absent in the otolithic end organs of αCGRP null (-/-) mice while choline acetyltransferase (ChAT) immunolabeling appeared unchanged suggesting the overall gross development of efferent innervation in otolithic organs was unaltered. Otolithic function was assessed by quantifying the thresholds, suprathreshold amplitudes, and latencies of vestibular sensory-evoked potentials (VsEPs) while general balance function was assessed using a modified rotarod assay. The loss of αCGRP in null (-/-) mice was associated with: (1) shorter VsEP latencies without a concomitant change in amplitude or thresholds, and (2) deficits in the rotarod balance assay. Our findings show that CGRP loss results in faster otolith afferent activation timing, suggesting that the CGRP component of the efferent vestibular system (EVS) also plays a role in otolithic organ dynamics, which when coupled with reduced VOR gain efficacy, impairs balance.

## Introduction

**FIGURE 1:**
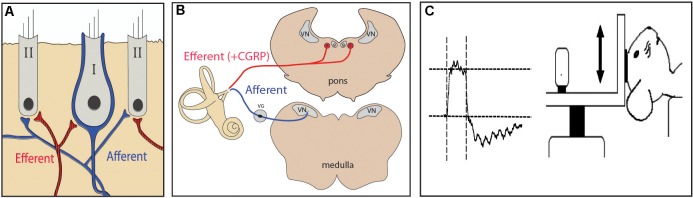
**(A)** Efferent neurons synapse directly onto calyceal afferents innervating vestibular type I hair cells. **(B)** Vestibular afferents project into the brainstem and synapse in the vestibular nuclei. Efferent cell bodies are found in the brainstem medial to the vestibular nuclei. **(C)** For otolith testing using vestibular evoked potentials (VsEP), a linear jerk pulse is applied to the mouse’s head via a mechanical shaker that moves the head along the naso-occipital axis.

Calcitonin gene-related peptide (CGRP) is a 37 amino-acid neuroactive peptide commonly found in efferent neurons innervating hair cell organs including the cochlea, lateral line, semicircular canals, and otolithic end organs (Adams et al., 1987; Wackym et al., 1993; Maison et al., 2003a,b; Dickerson et al., 2016). In mammals (**Figures 1A,B**), the efferent vestibular system (EVS) begins as hundreds of parent neurons in the dorsal brainstem, which project bilaterally and then branch extensively to make numerous synapses on type II hair cells and afferent terminals including calyces surrounding type I hair cells (Lysakowski et al., 1995; Holt et al., 2006; Lysakowski and Goldberg, 2008). At these synapses, CGRP is commonly colocalized in presynaptic efferent varicosities with choline acetyltransferase (ChAT), the enzyme that catalyzes the synthesis of acetylcholine (ACh), the predominant EVS transmitter (Goldberg, 2000). Electrical stimulation of the EVS produces excitatory afferent responses comprised of fast and/or slow components which are of larger amplitude in irregularly-discharging afferents (Goldberg and Fernandez, 1977; Goldberg et al., 1987; Curthoys et al., 1995). Similar efferent-mediated effects are evoked by high-velocity stimulation (Sadeghi et al., 2007, 2009). Recent pharmacological evidence has demonstrated a role for alpha9-containing nicotinic ACh receptors (α9 nAChRs) on type II hair cells and alpha4beta2-containing nAChRs and muscarinic AChRs on bouton and calyx-bearing afferents (Holt et al., 2015, 2017; Poppi et al., 2018).

However, the widespread co-expression of CGRP at these same cholinergic synapses suggests that it too impacts peripheral vestibular function (Guth and Norris, 1993; Guth et al., 1998; Holt et al., 2011). We have previously examined a transgenic mouse line [CGRP null (-/-)] with targeted deletion of the alpha isoform of the CGRP gene (αCGRP) where CGRP staining was clearly absent in the semicircular canal cristae, while ChAT immunostaining in the same tissue was comparable to cristae from control animals. The loss of αCGRP in null (-/-) mice was associated with a 50% decrease in vestibulo–ocular reflex (VOR) gain efficacy (Luebke et al., 2014). While a potential role for CGRP in semicircular canal function was identified, the significance of CGRP for otolithic organ function was unknown. We wondered if otolithic function in null (-/-) mice might also be affected by the loss of CGRP. Like canal cristae, we confirmed that CGRP staining was absent in the vestibular otolith end organs of CGRP null (-/-) mice, while ChAT staining in otolith end organs was comparable to wildtype mice. To access the functional implications of CGRP loss in the otolith end organs, we characterized the otolith-driven vestibular evoked potentials (VsEPs). VsEP measurements are altered in mice lacking α9 nAChRs (Morley et al., 2017), and we reasoned that the VsEP might also provide some insight into otolithic function in CGRP null (-/-) mice. In mice lacking CGRP, we found significantly shorter VsEP latencies without concomitant changes in VsEP amplitudes or thresholds. We further assessed balance function using a modified rotarod paradigm where we found that the performance of CGRP null (-/-) mice during testing was impaired compared to littermate controls. Collectively, our results suggest that the EVS neurotransmitter CGRP plays a role in tuning otolith dynamics that when coupled with reduced VOR gain efficacy impairs balance.

## Materials and Methods

### Animals

αCGRP null (-/-) and wildtype (+/+) transgenic mouse lines were created and characterized by the Emeson laboratory on a pure 129SvEv background (Lu et al., 1999). These animals were shipped to the Luebke laboratory, maintained as heterozygotes, and genotyped using their previously described methods. The αCGRP null (-/-) mice have a targeted deletion of αCGRP, produced by tissue-specific alternative splicing of the calcitonin/αCGRP gene while leaving the highly homologous βCGRP gene intact (Lu et al., 1999). Because the absence of αCGRP in the vasculature of CGRP (-/-) null animals was compensated by βCGRP, there were no reported abnormalities in heart rate or blood pressure under basal or exercise-induced conditions, which could have been confounding issues in our study.

αCGRP (+/-) mice were paired to generate homozygous CGRP (-/-) and CGRP (+/+) offspring used for all mouse studies. A total of 67 mice (both sexes) were used in these studies: *n* = 8 (+/+) and *n* = 18 CGRP (-/-) mice for VsEP testing, *n* = 3 (+/+) and *n* = 3 (-/-) mice for histology, and *n* = 20 (+/+) and *n* = 15 (-/-) mice were for rotarod performance. No sex differences in VsEP responses, rotarod balance function, or histology were found, so data were pooled across sex. Genotyped CGRP mice were shipped to the University of Nebraska for use in VsEP experiments. The care and use of these animals was approved by both University of Rochester’s and University of Nebraska’s Institutional Animal Care and Use Committees.

### Morphological/Immunohistochemical (IHC) Studies

Six mice, three each of αCGRP (-/-) and three αCGRP (+/+) (2M/1F) mice were anesthetized with ketamine (80 mg/kg)/xylazine (5 mg/kg) and perfused with heparinized PBS followed by 4% paraformaldehyde (PFA). Each animal was subsequently decapitated and the head was postfixed in 4% PFA overnight at 4°C. Whole otolithic maculae were extracted from the temporal bone and washed several times in 0.1 M phosphate buffer (PB). Maculae were then blocked with 5% normal donkey sera (Jackson Immunoresearch, Westgrove, PA, United States) and 0.5% Triton X in PB, and then incubated with primary antibodies (see below) in PB overnight. Following several washes with PB, tissue sections were reacted with the appropriate Alexa Fluor^®^-conjugated secondary antibodies (Molecular Probes/Invitrogen, Grand Island, NY, United States) at 1:500 in PB for 2–3 h. All tissues were then washed in PB, stained w/DAPI (10 μg/ml, Sigma, St. Louis, MO, United States), and mounted onto Plus Slides (Fisher Scientific) and coverslipped with SlowFade Gold mounting media (Invitrogen).

### Antibodies

Antibodies against ChAT and CGRP were used to label vestibular efferent fibers and varicosities in mouse otolithic organs. ChAT antibodies (Millipore, Billerica, MA, United States; AB144P lot JC1618187, 1:100 dilution or lot NG1780580, 1:250–1:500 dilution) were generated against the human placental enzyme. The CGRP (MU33) antibody (1:500 dilution) was generated against the amidated carboxyl seven amino acids of rat CGRP (Rosenblatt and Dickerson, 1997). These seven amino acids are conserved in mouse CGRP by both alpha- and beta-isoforms.

### Imaging/Quantification

Images of labeled efferent neurons in whole otolithic maculae were captured using either an Olympus FV1000 laser scanning confocal microscope (URMC Light Microscopy Core) with a PLAPON 60× oil objective and sequential scanning option or a Zeiss Axio Imager motorized upright multifluorescent microscope fitted with an Apotome slider system (Zeiss Imaging Systems, Oberkochen, Germany). Images and maximum intensity projections were created and post processed using Olympus FV1000 or Zeiss Axiovision imaging software. Adjustments of contrast and brightness were maintained within the linear range and Adobe Photoshop and Illustrator CS6 were used to compile final figures.

### VsEP Testing

For VsEP testing, 18 (8M/10F) αCGRP (-/-) and 8 (4M/4F) αCGRP (+/+) mice were anesthetized with an intraperitoneal injection of ketamine (90 mg/kg) and xylazine (10 mg/kg) mixture, and tested using VsEP recordings methods published previously (Mock et al., 2011). Linear acceleration pulses, 2 ms duration, were presented to the cranium via a non-invasive spring clip that encircled the head and secured it to a voltage-controlled mechanical shaker as shown in **Figure 1C**. Stimuli were presented along the naso-occipital axis at a rate of 17 pulses/sec. Stimulus levels ranged from +6 dB to –18 dB re: 1.0g/ms (where 1g = 9.8 m/s^2^) adjusted in 3 dB steps. Stainless steel wire was placed subcutaneously at the nuchal crest to serve as the non-inverting electrode. Needle electrodes were placed posterior to the left pinna and at the hip for inverting and ground electrodes, respectively. Traditional signal averaging was used where ongoing electroencephalographic activity was amplified (200,000X), filtered (300 to 3000 Hz), and digitized (100 kHz sampling rate), with 256 primary responses averaged for each waveform. All responses were replicated. A broadband forward masker (50 to 50,000 Hz, 94 dB SPL) was presented during VsEP measurements to verify absence of cochlear responses (Jones and Jones, 1999). VsEP thresholds were defined as the stimulus level midway between the minimum level that produced a discernable response and the maximum level where no response was detectable. Response peak latency was defined as the time, in milliseconds, from onset of the stimulus to the appearance of each positive (p1, p2) and negative (n1) response peak. Peak-to-peak amplitude (p1–n1) was measured in microvolts (μV). Thresholds, latencies, and amplitudes were compared between CGRP (-/-) null and CGRP (+/+) wild type controls using multivariate analysis of variance (MANOVA) and Student’s *t*-tests.

### Rotarod Testing

For modified rotarod testing 15 (8M/7F) αCGRP (-/-) and 20 (10M/10F) αCGRP (+/+) were testing using a Rotomex-5 Accelerating Rotarod (Columbus Instruments) using cylindrical dowels (69.5 mm in diameter). By using larger dowels (rat sized), the mice were effectively unable to grasp dowels and, as such, the rotarod becomes more of a test of balance and gait as detailed in Tung et al. (2014, 2016) . Rotarod testing occurred in three phases: Mice were first placed on a stationary rotarod for up to 60 s or until falling, whichever occurred first. Latency to fall was recorded automatically via a pressure sensitive floor panel. A second set of training trials was repeated for each mouse approximately 10 min after completion of the first set of stationary testing. Here, mice were placed first on a 5-rpm rotating dowel followed by a series of rotations that accelerated from 5 rpm to a final speed of 44 rpm over 60 s. During this time, mice were required to walk in a forward direction on the rotating dowels for as long as possible. When mice were no longer able to walk on the rotating dowels, they fell onto the landing platform below, which triggered the end of the trial for an animal. Training runs were repeated and measurements of time to fall (TTF) were noted for each trial. A final set of three rotation test trials were performed and TTF values were logged ∼ 15 min after the training runs. In this final phase, each test trial was separated by 10 min where mice were returned to cage for food and water and rest.

### Data Analysis

Graphpad Prism software (Version 7) was used for statistical analysis. Unless otherwise stated, analyzes were conducted using either two-tailed Student’s *t*-test or ANOVA, or their non-parametric equivalent, and significance was set at *p* < 0.05. Either Tukey or Bonferroni *post hoc* tests were used for multiple comparisons. Values are reported as mean ± SD unless noted otherwise.

## Results

**FIGURE 2:**
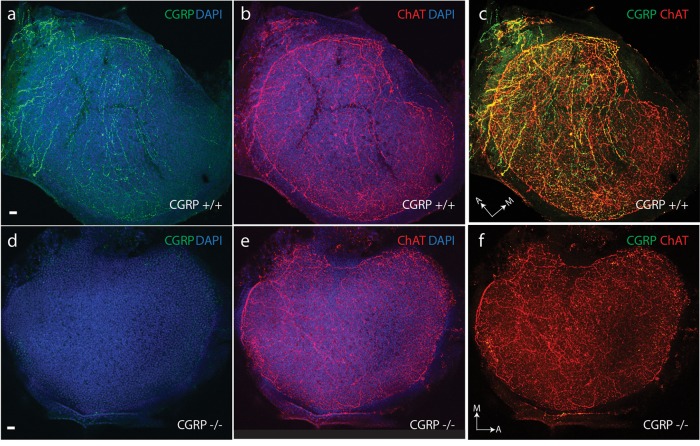
CGRP is abundantly present in the neuroepithelium of CGRP (+/+) mouse otolithic organs **(a)** with a distribution overlapping choline acetyltransferase (ChAT) expression **(b)** as illustrated in the composite image **(c)**. **(d)** CGRP is absent in the neuroepithelium of CGRP (–/–) mouse otolithic organs, while choline acetyltransferase (ChAT) expression **(e)** is comparable to that seen in the wildtype controls **(b)**. **(f)** Composite image for CGRP (–/–) otolith showing only ChAT staining. Staining for CGRP (green) and choline acetyltransferase-ChAT (red) with overlap indicated in yellow and orange. Arrows with letter labels indicate the anatomical orientation of the medial (M) and anterior (A) axes of the utricular maculae. Scale bars = 10 μm.

A light-microscopic examination revealed no gross structural abnormalities in the otolithic end organs of αCGRP (-/-) null animals. Utricles and saccules from αCGRP (+/+) and (-/-) animals were immunostained for CGRP and ChAT to visualize cholinergic efferent innervation, and were visualized using confocal microscopy. In the utricular macula of CGRP (+/+) mice, antibodies to CGRP and ChAT strongly labeled efferent fibers and varicosities (**Figures 2a,b**). There was an extensive overlap in the distributions of staining for CGRP and ChAT where immunoreactive varicosities positive for both ChAT and CGRP appear as yellow and orange puncta (**Figure 2c**). There were some variations in the labeling intensity for ChAT and CGRP across the macula but no consistent patterns were observed that might indicate differences in regional innervation. Inspection at higher magnification revealed that most CGRP neurons were also ChAT-positive. However, there were short strings of fibers and variosities that appeared to primarily express CGRP. While we cannot exclude the possibility that these minor innervation patterns represent a distinct neuronal population, subsequent varicosities along the same string were immunostained for both ChAT and CGRP suggesting, instead, that the content of individual varicosities may vary (**Figure 2c**). Inspections at higher magnification, however, revealed that most fibers and varicosities were positive for both. Whether these represent distinct efferent neuronal groups is unclear. Comparable immunohistochemistry in αCGRP (-/-) mice revealed that CGRP immunoreactive fibers and varicosities were absent from utricular epithelium (**Figure 2d**). When images from CGRP and ChAT immunostaining in CGRP (-/-) mice were merged, only ChAT+ fibers and varicosities were detected (**Figures 2e,f**). Despite the loss of CGRP staining, ChAT labeling appeared similar between CGRP (+/+) and CGRP (-/-) mice (**Figures 2b,e**). Comparable observations were made in saccular maculae (data not shown).

The VsEP is a sensitive measure to characterize the functional status of the otolith organs (Jones and Jones, 1999, 2014; Jones et al., 2001; Brown et al., 2017). A concise and definitive summary of the physiological basis of the VsEP and the nature of the vestibular neurons generating the VsEP can be found in Jones et al. (2015).

VsEPs are compound action potentials generated when a linear jerk pulse is applied to the mouse’s head (**Figure 1C**). The responses can be recorded from the surface of the skull much like the widely used auditory brainstem response (ABR), and like the ABR, the VsEP contains one response peak that is generated by the peripheral vestibular nerve (p1–n1) and two or more peaks generated by central vestibular relays (p2 and beyond). To test the effects that the loss of CGRP would have on otolithic function, we recorded VsEPs in mice lacking CGRP (-/-) and control (+/+) mice.

**FIGURE 3:**
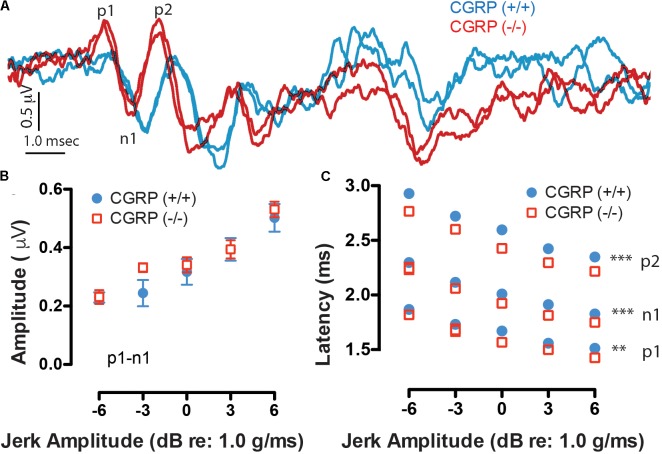
**(A)** Robust duplicative VsEP waveforms were collected having two to three response peaks (p1, n1, and p2 are labeled), as shown to a 6 dB jerk amplitude (relative to 1.0 g/ms). Waveforms at varying stimulus levels were obtained and then quantified in terms of amplitude **(B)** and latency **(C)** as a function of jerk stimulus level. Here, CGRP (–/–) mice (red) show similar waveform morphology and peak amplitudes as CGRP (+/+) mice (blue) but have significantly prolonged peak latencies suggesting timing differences in neural activation, ^∗∗^*p* < 0.005, ^∗∗∗^*p* < 0.001.

Robust VsEPs were obtained for both CGRP (+/+) and CGRP (-/-) mice. Representative waveforms for CGRP (+/+) and CGRP (-/-) mice are shown as blue and red traces in **Figure 3A**, respectively. VsEP thresholds for CGRP (+/+) mice ranged from -4.5 to -13.5 dB re: 1.0 g/ms and averaged -9.0 ± 2.8 dB re: 1.0 g/ms. The threshold for CGRP (-/-) mice ranged from -1.5 to -10.5 dB re: 1.0 g/ms with a mean of -7.1 ± 2.8 dB re: 1.0 g/ms. On average, there was no significant difference in VsEP thresholds between the CGRP (+/+) and CGRP (-/-) null mice. Response amplitudes (p1–n1), which are reflective of the population and synchrony of primary afferents responding to the stimulus, were also similar between CGRP (+/+) and CGRP (-/-) null animals (**Figure 3B**). However, response peak latencies (p1 and n1), reflecting activation timing of the primary afferents, were shorter for the CGRP (-/-) null mice at all stimulus levels (**Figure 3C**). MANOVA completed for data at +6 dB demonstrated that the latency differences were significant for p1 (*p* = 0.004), n1 (*p* = 0.014), and p2 (*p* = 0.006). *Post hoc* Tukey analysis showed significant latency differences at all jerk levels p1 (*p* = 0.0028), n1 (*p* = 0.0009), and p2 (*p* = 0.0001). Yet, p1–n1 amplitude MANOVA or *post hoc* testing revealed no significant differences between CGRP (-/-) null and (+/+) wildtype controls.

**FIGURE 4:**
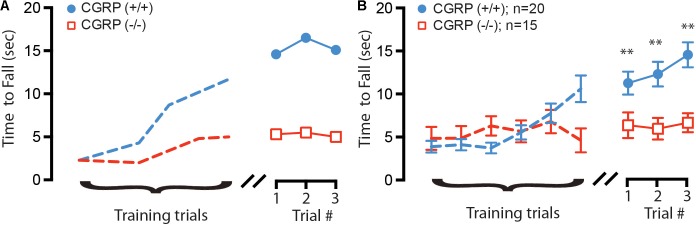
**(A)** Example training (dashed lines) and testing trials for two 2 month (age-matched) female mice, with CGRP (–/–) null (red) and CGRP (+/+) control (blue) mice. **(B)** Composite training and testing trials for all mice, with CGRP (–/–) null (red, *n* = 20) mice fall off rotating rotarod on average 6.4 s sooner than CGRP (+/+) control (blue, *n* = 15) mice, ^∗∗^*p* < 0.005.

We further asked whether the changes in VOR efficacy (Luebke et al., 2014) and VsEP response latencies (this study) might impact balance function in CGRP (-/-) mice. To evaluate if differences in general balance performance existed between CGRP (+/+) and CGRP (-/-) mice, we performed rotarod testing. To test gross balance function and acclimate the mice to the behavioral task, both CGRP (+/+) control and CGRP (-/-) null mice were placed on a stationary rotarod and the total time spent on the rod before falling was recorded up to a maximum of 60 s. Both CGRP (+/+) and CGRP (-/-) mice were able to balance on a stationary rotarod for the full 60 s (data not shown). We then performed six training runs on a slowly accelerating rotarod. While both CGRP (+/+) and CGRP (-/-) mice were able to learn the task (**Figure 4A**), the CGRP (+/+) mice showed steeper improvement. However, on average, the CGRP (-/-) mice were significantly impaired relative to CGRP (+/+) controls (*p* = 0.003) in all three testing trials (**Figure 4B**). When all three trials were averaged for CGRP (+/+) and CGRP (-/-) mice, the control (+/+) mice were able to stay on the rotating rotarod 6.4 s longer than (-/-) mice.

## Discussion

In this study, we show that mice with a targeted deletion of the αCGRP gene display a decrease in otolith activation timing and balance impairments. Specifically, the loss of αCGRP in (-/-) null mice was linked to a significant shortening of the VsEP latency without concomitant changes in thresholds or amplitudes. Immunohistochemistry confirmed that CGRP was absent in the otolith organs of null (-/-) mice, while ChAT staining appeared normal suggesting that the gross development of EVS innervation patterns was unaltered. Lack of changes in VsEP amplitudes and thresholds suggests that the general sensitivity and neural synchrony of the activated otolithic neurons are also likely unaffected in αCGRP (-/-) animals. Taken together, the VsEP results suggest that the absence of CGRP enhances activation timing of vestibular primary afferents. Our findings further suggest that the change in otolith response dynamics in αCGRP (-/-) animals, in conjunction with a reduced VOR gain efficacy (Luebke et al., 2014), may contribute to their impaired balance on a rotarod balance assay.

Our findings that loss of CGRP enhances otolithic activation timing suggest that CGRP normally slows the time for transduction currents and/or postsynaptic transmitter release to activate the trigger zone of primary afferents. Similar observations in VsEPs have been reported for α9 and α10 nAChR subunit knockout animals (Morley et al., 2017), yet in those studies, general sensitivity of the afferents (i.e., amplitude and threshold) was also affected by the loss of the same nAChR subunits. Additionally, α9 nAChR subunit knockout animals are not able to modify their VOR after a unilateral labyrinthectomy, yet exhibit normal baseline VOR gains (Hubner et al., 2015, 2017). Interestingly, the loss of CGRP loss in the semicircular canals results in 50% decreased VOR gain (Luebke et al., 2014), although it is not known if the loss of CGRP impedes VOR compensation. Given CGRP’s loss in the semicircular canals results in decreased VOR gain (Luebke et al., 2014), and CGRP’s loss in otoliths results in faster activation timing; CGRP released by EVS neurons onto the vestibular end organs may therefore serve as an activation control mechanism that could ultimately adjust temporal and gain aspects of motor reflexes which could result in balance impairments, as we have observed using the modified rotarod assay (Tung et al., 2014, 2016).

However, while CGRP loss is most striking in the otolith periphery, CGRP-dependent effects in central vestibular circuitry may also contribute to balance impairments observed; as CGRP is present in some vestibular nuclei cells that project to the EVS and may likely regulate its function (Wackym et al., 1993; Chi et al., 2007; Ahn et al., 2009). Moreover, CGRP loss from birth may also impair upstream mechanisms that mediate balance due to changes to the peripheral and/or central pathways. It is also possible that CGRP-dependent modulation influences vestibular pathways over a shorter time scale. For instance, increased CGRP levels have been linked to migraine (Villalon and Olesen, 2009), and migraine can often by accompanied by vertigo, dizziness, nausea, and sometimes by abnormal caloric or vestibular-evoked myogenic potentials (Szirmai, 1997; Furman and Marcus, 2012; Furman et al., 2013; Lempert et al., 2013; Furman and Balaban, 2015). While we have observed rotarod balance deficits with the complete loss of CGRP, right/left imbalances between CGRP signaling in migraine feedback could also contribute to migraine-associated vertigo and balance disturbances. In fact a recent report suggests that CGRP-induced fluctuations in afferent responses may also contribute to the generation of motion sickness (Xiaocheng et al., 2012). Future experiments, aimed at understanding the effects of CGRP signaling imbalances may be required to fully elucidate CGRP’s role in mediating vestibular function.

## Author Contributions

AL, SJ, JH, PJ, SD, and SV conceived and designed the experiments, and participated in the acquisition, analysis, and interpretation of the experimental findings. AL, SJ, PJ, and JH wrote and revised the manuscript. SJ, JH, and AL gave the final approval of the manuscript and are accountable to all aspects of the work.

## Conflict of Interest Statement

The authors declare that the research was conducted in the absence of any commercial or financial relationships that could be construed as a potential conflict of interest.
